# Crack detection in structural images using a hybrid Swin Transformer and enhanced features representation block

**DOI:** 10.3389/frai.2025.1655091

**Published:** 2025-12-01

**Authors:** N. Anusha, L. Jani Anbarasi

**Affiliations:** 1 Department of IoT, School of Computer Science and Engineering, Vellore Institute of Technology, Vellore, India; 2 School of Computer Science and Engineering, Vellore Institute of Technology, Chennai, India

**Keywords:** swin transformer, crack detection, convolutional neural network, residual network, population-based optimization

## Abstract

**Introduction:**

This paper presents a crack detection framework employing a hybrid model that integrates the Swin Transformer with an Enhanced Features Representation Block (EFRB) to precisely detect cracks in images.

**Methods:**

The Swin Transformer captures long-range dependencies and efficiently processes complex images, forming the backbone of the feature extraction process. The EFRB improved spatial granularity through depthwise convolutions, that focus on spatial features independently across each channel, and pointwise convolutions to improve channel representation. The proposed model used residual connections to enable deeper networks to overcome vanishing gradient problem.

**Results and discussion:**

The training process is optimized using population-based feature selection, resulting in robust performance. The network is trained on a dataset split into 80% training and 20% testing, with a learning rate of 1e-3, batch size of 16, and 30 epochs. Evaluation results show that the model achieves an accuracy of 98%, with precision, recall, and F1-scores as 0.97, 0.99, and 0.98 for crack detection, respectively. These results show the effectiveness of the proposed architecture for real-world crack detection applications in structural monitoring.

## 1 Introduction

Machine vision technology has seen significant advancement in the field of road crack detection (Yin et al., 2023). Image and video analysis demonstrate a remarkable capacity for detecting and identifying early signs of road cracks. They play a crucial role in monitoring and early warning, helping to prevent potential crack-related incidents and safeguard lives and property (Ma and Mei, 2021). Traditional pavement crack detection methods include techniques like minimum-path algorithms, image thresholding, and wavelet transformations. To enhance accuracy, few methods integrate free-form anisotropy and morphological filters, to attain a clearer depiction of crack intensity and features. Also, collaborative crack detection techniques have employed Sobel edge detectors with two-dimensional empirical mode decomposition, improving the precision of surface crack differentiation. Convolutional Neural Networks (CNN) are used for analyzing pavement crack images and surface characteristics to effectively enhance crack identification accuracy.

Cracks in road surfaces are common during road construction, initially arising from material aging and degradation over time and also due to climatic factors like precipitation and snow. These elements result various types of surface cracks, which gradually expand and lead to both surface and structural deterioration, compromising road safety and durability. In recent decades, various researchers and experts have proposed multiple techniques for detecting cracks in road surfaces, including physical inspection, machine vision, and infrared imaging. Physical inspections are intensive, inefficient, and prone to human errors (Zhao et al., 2025). Machine vision techniques allows automated detection but needs high-quality image data and algorithms. Infrared imaging analyse the temperature on road surfaces that may indicate cracks but involves substantial equipment costs (Oloufa et al., 2004; Liu et al., 2024).

Recently, deep learning has significant importance in image processing, leading to the extensive use for road surface crack detection due to their high efficacy. Identifying and detecting structural surface issues, particularly cracks, can offer consistent data for the maintenance of buildings. Traditional crack detection methods, often produce subjective results (Zhao et al., 2022) and lack a standardized global framework, reducing accuracy. Advances in computer vision have resulted in crack detection algorithms that offer automation, efficiency, and non-contact capabilities, effectively addressing the limitations of manual methods. In particular, the rapid progress of deep learning technology in recent years has enabled CNN models to significantly enhance detection accuracy and efficiency. Currently, CNN based detection methods are applied to identify surface damage in buildings, bridges, and tunnels. The image classification schemes identify the category of the input image, the object detection model estimates object locations within the image, and the semantic segmentation model performs pixel-level analysis to pinpoint objects. While semantic segmentation provides the highest accuracy, it requires pixel based labelled data for training, which is difficult and limits the CNN models in the field of structural crack detection. The main contribution of the proposed work is as follows:

- A Hybrid framework integrating the Swin Transformer with an Enhanced Features Representation Block (EFRB) to capture both global dependencies and fine-grained spatial features for accurate crack detection.
- Depthwise and pointwise convolutions in the EFRB enhanced spatial granularity and channel-wise representation while residual connections and normalization stabilized the training and prevent overfitting.
- Population-based feature selection with adaptive optimization further refines discriminative features, reducing computational complexity thus improving model generalization.
- This approach outperforms, achieving 98% accuracy with better precision, recall, and F1-scores.

The paper is organized as follows: Section 2 details the review of existing crack detection methodologies. Section 3 presents the proposed hybrid Swin Transformer with Enhanced Features Representation Block (EFRB) and population-based feature optimization approach. Section 4 describes the dataset, evaluation metrics, ablation studies, and performance analysis of the proposed model. Section 5 concludes the paper.

## 2 Related work

Nguyen et al. (2021) proposed a two-stage CNN where the first stage reduces noise and isolates potential cracks, while the second stage focuses on learning contextual features of cracks within the identified areas. The DeepCrack dataset, used for detection and segmentation validation, includes 537 images of 544 × 384 pixels with pixel-level ground truth annotations (Liu et al., 2019). Another dataset, CrackIT (Oliveira and Correia, 2014), was compiled in Portugal and Canada for crack analysis. Fan et al. (2020) introduced an automated crack detection system using a U-Hierarchical Dilated Network (U-HDN). This model uses hierarchical feature learning and dilated convolution for detailed crack detection on road pavements. By integrating multiple context sizes through a multi-dilation module, the U-HDN model improves its capability to capture complex crack patterns at various scales. Tests on public crack datasets show that U-HDN outperforms existing methods by effectively combining diverse context sizes and multi-scale feature maps, leading to an increased detection accuracy of 0.93. Liu et al. (2022a) leveraged deep learning, specifically CNNs, and infrared thermography to categorize asphalt pavement crack into four categories: no crack, low, medium, and high severity. Results showed fusion images resulted the good accuracy for models built from scratch, while visible images performed best in transfer learning, with EfficientNet-B3 achieving the highest accuracy across all categories for both methods.

Elghaish et al. (2022) evaluated models like AlexNet, GoogleNet, and two others for highway crack identification and classification, and introduced a new CNN model optimized for accuracy across diverse learning rates. The novel CNN model achieved 97.62% accuracy using a dataset of 4,663 crack images grouped into three categories, outperforming GoogleNet’s 89.08% and AlexNet’s 87.82%, utilizing Adam optimization at a learning rate of 0.001 for efficient highway crack recognition. Ahmadi et al. (2022) proposed a approach combining segmentation, noise reduction, heuristic-based feature extraction, and the Hough transform with crack classification using six classifiers. The hybrid model achieved the highest accuracy at 93.86%, surpassing individual classifiers. Liu et al. (2022b) employed infrared thermography and CNNs to classify asphalt pavement fatigue crack severity into four levels, using three image types. CNN models, including EfficientNet-B4, were trained, with accuracy surpassing 0.95 across all image types, particularly on infrared images. Grad-CAM and Guided Grad-CAM analyses indicated fusion images are highly effective for reliable fatigue crack classification.

Oliveira and Correia (2014) developed a comprehensive MATLAB toolbox for crack detection and characterisation on road pavement surfaces, which includes algorithms for preprocessing, crack identification, and classification. The toolbox includes 84 pavement surface images obtained from standard road surveys, providing a valuable resource for evaluating crack detection algorithms. Yamaguchi and Hashimoto (2010) presented a rapid crack identification method for concrete surfaces using percolation-based image processing, which reduces computational time by incorporating skip processes and assessing pixel circularity. Experimental results show reduced computation costs while maintaining high crack detection accuracy. Vivekananthan et al. (2023) developed a grey intensity adjustment model for crack detection, employing grey level discrimination and the Otsu method to set threshold ranges and Sobel’s filter for edge detection. This approach achieved a maximum detection accuracy of 95% while addressing constraints related to aspect ratio and margin parameters. Liu et al. (2023) introduced a tunnel crack detection method using image processing with deep learning, comparing SVM and AlexNet based models. AlexNet achieved 96.7% test accuracy, indicating deep CNN models’ superior performance for identifying structural flaws in subway tunnels. Malek et al. (2023) created a real-time augmented reality (AR) crack detection system, overcoming traditional AR limitations by adapting the Canny algorithm to the AR headset platform for autonomous processing. Experimental results confirm this AR method’s efficiency and practicality for real-time crack detection in field inspections. Tran et al. (2023) presented a process based deep learning approach for bridge deck crack detection and segmentation, testing five object detection networks including YOLOv7 and achieved a detection accuracy of 92.38%. The proposed U-Net also exhibited enhanced performance, successfully identifying and quantifying cracks on bridge decks.

Pham et al. (2023) employed U-Net, LinkNet, FPN, and Deeplabv3, achieving F1 scores between 0.877 and 0.896 at 7.48–8.01 frames per second (FPS), notably outperforming traditional image processing methods in speed and accuracy. Nyathi et al. (2023) developed an approach for measuring concrete crack, achieving high precision with absolute error between 0.02 mm and 0.57 mm, facilitating compliance with international standards. Zhang et al. (2023b) presented a lightweight crack detection technique for bridges using YOLOv4, incorporating lighter networks to reduce computational demands for edge devices. The modified YOLO v4 achieved 93.96% precision, 90.12% recall, and F1 score of 92%, requiring only 23.4 MB and running at 140.2 FPS. Xu et al. (2023) introduced the YOLOv5-IDS model, integrating the YOLOv5 architecture with a bilateral segmentation network for concrete crack detection and measurement, achieving an mAP@0.5 of 84.33% and an mIoU of 94.78%, with rapid detection at 159 FPS.

Hu et al. (2024) proposed an advanced approach to road surface crack detection using an enhanced YOLOv5 model, addressing the complexities of information extraction from vehicle-mounted imagery. Key improvements include the Slim-Neck architecture for targeted crack focus, the C2f structure and Decoupled Head for optimized data utilization, and a split SPPCSPC structure for enhanced efficiency and precision. Experimental results demonstrate significant improvements across multiple evaluation metrics compared to five other sophisticated models, affirming the effectiveness of the proposed approach. Dong et al. (2024) introduced YOLOv8-Crack Detection (YOLOv8-CD), a lightweight, optimized algorithm for concrete crack detection aimed at boosting infrastructure safety and maintenance efficiency. The model leverages visual attention networks and a Large Separable Kernel Attention module to enhance crack shape detection and feature extraction. Experimental findings reveal substantial gains in mAP scores and detection speed, achieving 88 FPS while reducing processing demands, thereby validating its advantage over other object detection techniques. Chen et al. (2023) explored the role of deep learning, specifically transfer learning, in automating the detection of building cracks. Addressing the need for efficient large-scale inspections, transfer learning significantly improved CNN performance, boosting accuracy from 89 to 94%, demonstrating its efficacy in image classification with limited data, aligning with national smart nation goals for intelligent technology in construction.

Zhang et al. (2023a) present a lightweight learning model for concrete crack detection, named MobileNetV3-BLS, which overcomes the challenges of complex architectures and high computational requirements. This method improves feature extraction by integrating MobileNetV3’s inverted residual structure as a convolutional module, employing random mapping and enhancement nodes to train the model. MobileNetV3-BLS exhibits enhanced accuracy and training speed, facilitating dynamic updates for incremental learning with new data and nodes. Zadeh et al. (2024) evaluate multiple deep learning architectures: InceptionV3, VGG19, ResNet50, and EfficientNetV2 using fine-tuning for concrete crack detection. Results show EfficientNetV2 achieves 99.6% accuracy, 99.3% precision, and a recall of 1, leading to a balanced F1 score of 99.6%, effectively minimizing false positives and maximizing true crack identification.

Guo F. et al. (2024) analysed in two stages where Stage I detects images with pixel cracks using a CNN-based classifier, while Stage II uses a separation combination approach and CTv2 (Crack Transformer v2) for pixel level detection. Extensive testing confirms the framework’s advantage and efficiency, facilitating scalable automated pavement crack detection. Karimi et al. (2024) developed a robust deep learning model for detecting cracks across various Cultural Heritage (CH) materials using the YOLO object detection network. The study examines masonry types (stone, brick, cob, tile) and modern materials like concrete with a dataset of 1,213 images across categories. Results show mean average precision values of 94.4% for concrete, 93.9% for concrete and cob, 92.7% for cob, 87.2% for stone, 83.4% for stone and brick, 81.6% for brick, and 70.3% for tile, highlighting the model’s potential for efficient CH crack detection, aiding specialists in damage assessment.

Guo C. et al. (2024) proposed SegCrackNet, an innovative neural network with multi-level output fusion, dropout layers, and T-bridge block configurations to reduce overfitting and enhance the utilization of contextual information. Experimental results reveal notable improvements over other models, with IoU score increases of 4.3%, 9.4%, and 3.7% for the Crack500, Crack200, and pavement images datasets, respectively. Yu et al. (2024) presented an optimized lightweight segmentation model similar to BiSeNetv for automated pavement crack detection. Results show that this model outperforms prior methods with an F1 score improvement of 10.14%, underscoring its precision and robustness in segmenting pavement cracks.

Liu and Xu (2023) utilized a VGG16-based CNN for crack classification, incorporating an enhanced Class Activation Map (CAM) technique for precise localization and distribution of cracks. Integrating simple linear iterative clustering (SLIC) superpixel segmentation with CAM, the semantic segmentation accuracy is improved to a greater extend. Bayesian optimization identifies ideal parameters, and test results that indicate the algorithm’s support on image-level labelling that significantly reduced labour and cost thus maintaining accuracy. Table 1 details an overview of the various crack detection methods.

**Table 1:** An overview of different DL methodologies for crack detection.

Ref	Methodology	Categories	Dataset	Metric	Inference
Nguyen et al. (2021)	CNN	crack detection and segmentation	DeepCrack, CrackIT, 2StagesCrack dataset	F1-measure - 0.91	Achieves high performance on noisy, low-resolution, and imbalanced data.
Fan et al. (2020)	U-Hierarchical Dilated Network	Pavement crack detection	AigleRN	Pr: 0.92, Re: 0.93, F1: 0.92 accuracy – 93%	Superior detection accuracy through multi-scale feature extraction.
Liu et al. (2022a)	Transfer Learning Models	Pavement crack severity classification	Asphalt	Accuracy −93%	Fusion images yield better accuracy; EfficientNet-B3 performs best across all image types.
Elghaish et al. (2022)	convolutional neural network	Highway cracks	4,663 images of highway cracks	97.62% accuracy	New CNN model outperforms existing models like GoogleNet and AlexNet.
Ahmadi et al. (2022)	neural network, decision tree, SVM, KNN, Bagged Trees,	Crack classification	400 images	93.86% accuracy	Hybrid model surpasses individual classifiers for crack classification.
Liu et al. (2022b)	EfficientNet-B4	Pavement fatigue crack severity classification	2,211 images, while their size is 640 × 480. asphalt	Accuracy – 95%	Fusion images are effective for fatigue crack severity classification.
Oliveira and Correia (2014)	CrackIT toolbox algorithms	Crack detection	84 pavement surface	re = 98.4% pr = 95.5% 100% of recall	Toolbox provides crack detection and characterization algorithms for research use.
Yamaguchi and Hashimoto (2010)	Percolation-based image processing	Concrete crack detection	60 images concrete surfaces images	Pre-0.95	Reduced computation costs.
Vivekananthan et al. (2023)	Gray intensity adjustment model for crack detection		2068 crack images	95% detection accuracy	Otsu and Sobel methods improve crack detection accuracy.
Liu et al. (2023)	Image processing and deep learning and SVM	Crack images in subway tunnels,	3,000 data images	SVM: 88%; AlexNet: 96.7%	Performs effectively for crack detection in tunnels.
Tran et al. (2023)	Process-based deep learning for bridge deck crack detection	Crack detection and segmentation	Two bridge datasets l bridge decks in South Korea	Precision −0.83	Outperforms other networks in speed and accuracy for crack detection.
Pham et al. (2023)	U-Net, LinkNet, Feature Pyramid Network and Deeplabv3	Ground crack detection	510 crack, 185 slope and 325 field images	F1 score: 0.877–0.896	Outperform traditional methods for crack identification and measurement.
Zhang et al. (2023b)	YOLO v4	Bridge crack detection	About 800 photos of bridges around Guizhou University.	Precision: 93.96%; Recall: 90.12%	Method is effective for edge deployment with minimal computational requirements.
Xu et al. (2023)	YOLOv5-IDS	Crack detection and segmentation	302 crack images	mAP@0.5: 84.33%; mIoU: 94.78%	Achieves high accuracy and processing speed for crack detection.
Hu et al. (2024)	Improved YOLOv5	Road surface	13,508 images	F1 score – 0.5876	Enhance information extraction from vehicle-mounted images for better crack detection.
Dong et al. (2024)	YOLOv8-Crack Detection (YOLOv8-CD)	Crack detection	RDD2022 and Wall Crack datasets	precision of 91.5%	Improves feature extraction and detection speed for concrete surface cracks.
Chen et al. (2023)	Transfer learning for automated building facade crack inspection	Crack detection	3,600 building crack images	Traditional CNN: 89%; Transfer learning: 94%	Significantly enhances crack classification performance.
Zadeh et al. (2024)	Deep learning architectures	Surface crack detection and classification	20,000 images from structures within the METU Campus	InceptionV3–94%	Achieves high accuracy and minimizes false positives in crack detection.
Guo F. et al. (2024)	Two-stage framework CNN with a transformer model	Pavement surface crack detection	CrackSD dataset	Deep LabV3–97.21	Identifies and detects pixel-level cracks for large-scale applications.
Karimi et al. (2024)	YOLOv5	Crack damages	1,213 bricks	Mean AP: 94.4%	Identifies cracks in various materials, supporting inspection professionals in damage assessments.
Guo C. et al. (2024)	SegCrackNet for crack detection		Crack500, Crack200, and pavement images datasets	79.85, 44.97 and 49.66%	Effectively detects subtle variations and improves crack detection accuracy.
Yu et al. (2024)	BiSeNetv2	Pavement surface crack detection	CFD dataset, Crack500, CrackSC	Recall - 91.09	Demonstrates effectiveness and robustness in segmenting pavement surface cracks.

Shaoze et al. (2025) introduced a variant in YOLO achieving 86.4% mAP@50, demonstrating strong detection performance in complex environments. Tang et al. (2024) proposed BsS-YOLO for road crack detection, integrating improved PAN and BiFPN feature fusion structures along with attention mechanisms, leading to a 2.8% mAP gain over baseline YOLO models. For bridge crack detection, Dong et al. (2025) proposed YOLO11n-BD that incorporates an Efficient Multi-Scale Cross Attention (EMSCA) module and a Lightweight Dynamic Head (LDH), achieving 94.3% mAP@50 and an F1-score of 89.2% while maintaining real-time performance at 555 FPS. Zhu et al. (2025) proposed FD^2^-YOLO that enhanced YOLOv11n with a dual-stream architecture combining spatial and frequency-domain features, improving detection robustness on noisy surfaces with 88.3% mAP@50 and 88.4% precision. Zhao et al. (2025) developed a YOLOv11 for intelligent tunnel lining crack detection, achieving 93.3% accuracy, 94.5% recall, and 96.9% average precision. Their approach effectively identifies cracks under complex lighting and structural conditions, ensuring robust performance for real-world tunnel inspections.

## 3 Proposed methodology

**Figure 1:**
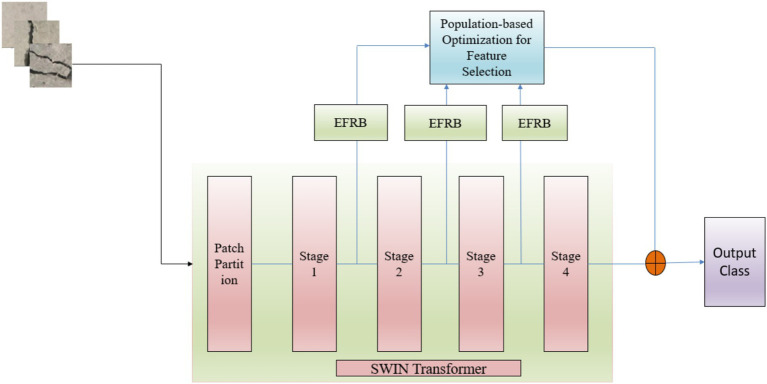
Architecture of the proposed system.

The proposed study presents custom hybrid framework for detecting surface cracks in concrete floors as illustrated in Figure 1. The main objective is to create a computationally efficient and accurate model that integrates the strengths of the Swin Transformer, skip learning, Enhanced Features Representation Block, along with an attention mechanism to precisely identify surface cracks in concrete structures.

This model used the Swin Transformer ( ${\mathrm{ST}}_{B})\;$ and skip connections, fine-tuned through an Enhanced Feature Representation Block ( ${\mathrm{EFR}}_{B})$ , with varying filter sizes. Feature selection and optimization are achieved using Population-Based Optimization ( ${\text{POFS}}_{B})$ which conducts a randomized search to identify optimal solutions for the efficient crack detection in images.

### 3.1 Swin Transformer

**Figure 2:**
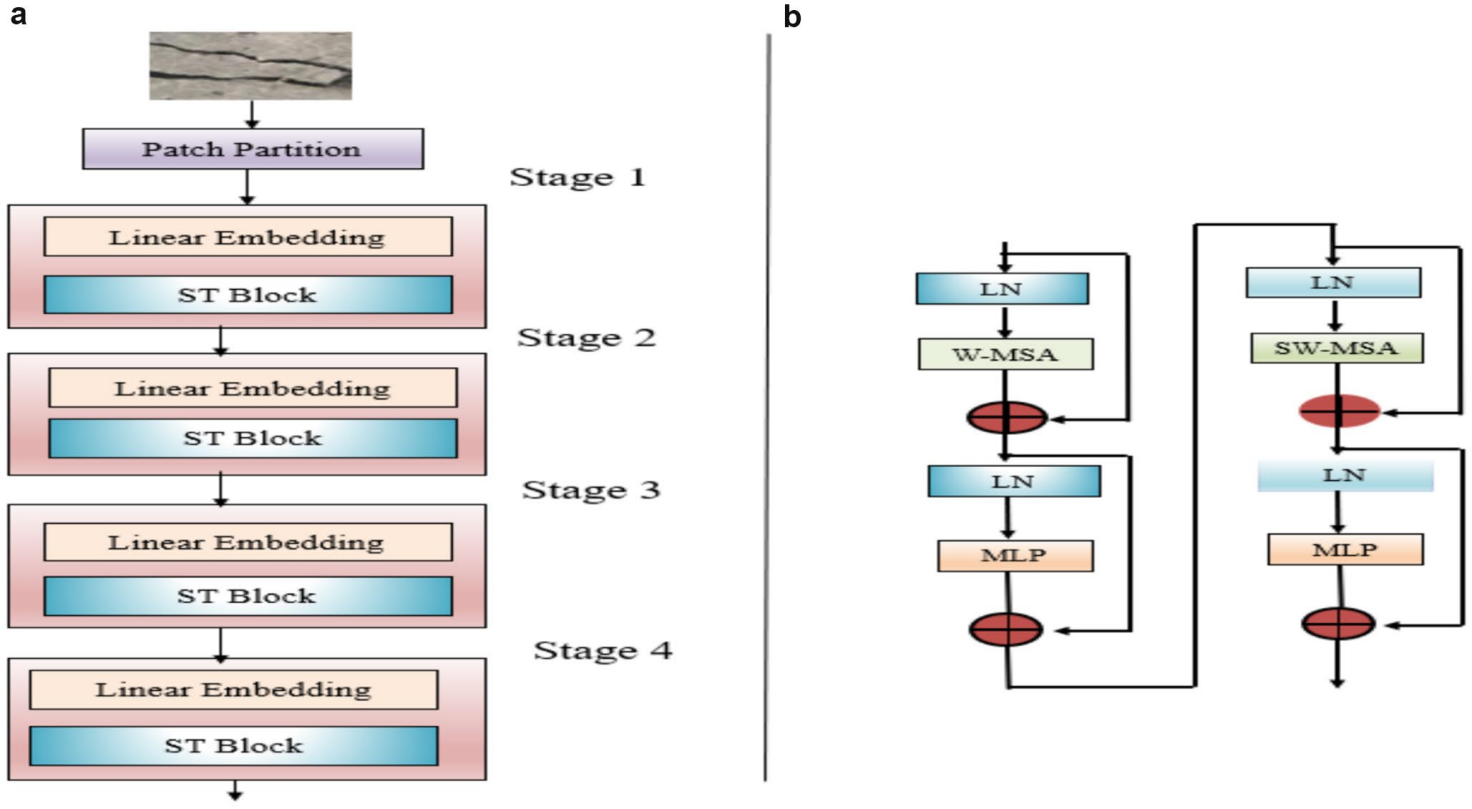
Flow of layers in Swin Transformer.

Unlike CNNs, Vision Transformers (ViTs) utilize the attention mechanism of Transformers for image data. A key benefit of ViT is its ability to represent global features without depending on local receptive fields. Transformers self-attention necessitates calculating weights between all other tokens, leading to increased computational complexity. As a result, the computational cost associated with super-resolution images can be substantial. In contrast to ViT, the Swin Transformer (Liu et al., 2021) incorporates a mechanism known as the shifted window, which segments into non-overlapping localized. Features are further processed among windows through this shifting process. Swin Transformer employed a hierarchical process composed of various stages, each containing several transformer blocks. Figure 2 provides the summary of the Swin Transformer architecture.

The input image, of size H×W×3 , is splitted into non-overlapping patches of size $\frac{H}{4}\times \frac{W}{4}\times 48$ . The input data is processed at the final stage through a linear layer that transforms the feature into C which is enhanced through an attention model. The same operations are repeated in the subsequent three stages. The adjacent 2×2 patches are combined through a patch merging, which reduces size by half through a linear layer followed by multiple blocks to enhance the merged patches using attention blocks. Ultimately, the resulting data has dimensions of $\frac{H}{32}\times \frac{W}{32}\times 8C$ . Figure 2 depicts two consecutive Swin Transformer blocks, where the conventional multi-head self-attention mechanism (MSA) is substituted with window-based multi-head self-attention (W-MSA) and shifted window multi-head self-attention (SW-MSA). By leveraging the partitioning shifted window technique, the representation generated by successive Swin Transformer blocks can be expressed as Equations 1–4:


$${\hat{s}}^{l}=W-\mathrm{MSA}\;(L_{N}(s^{l-1}))+s^{l-1}$$ (1)

$$s^{l}=\mathrm{MLP}\;(L_{N}({\hat{s}}^{l}))+{\hat{s}}^{l}$$ (2)

$${\hat{s}}^{l+1}=\mathrm{SW}-\mathrm{MSA}\;(L_{N}(s^{l}))+s^{l}$$ (3)

$$s^{l+1}=\mathrm{MLP}\;(L_{N}({\hat{s}}^{l+1}))+{\hat{s}}^{l+1}$$ (4)

Where ${\hat{s}}^{l},s^{l}$ represent the output of the (S)W-MSA and the MLP module of block 𝑙, respectively; and SW-MSA and W-MSA refer to window-based multi-head self-attention mechanisms that utilize standard and shifted window partitioning processes, respectively. Consider each window includes M×M patches, the complexity of multi-head self-attention module and W-MSA for h×w patches are as given in Equations 5, 6:


$$\Omega \mathrm{MSA}=4{\mathrm{hwC}}^{2}+2\;{\left(\mathrm{hw}\right)}^{2}C$$ (5)

$$\mathrm{\Omega W}-\mathrm{MSA}=4{\mathrm{hwC}}^{2}+2\;M^{2}\mathrm{hwC}$$ (6)

The complexity of MSA is quadratically linked to patch count, meaning it increases significantly with a larger number of patches. In contrast, when the size M is constant, the complexity of W-MSA remains linear. As a result, the rise in complexity is quite modest even with a greater number of patches. This characteristic improves the scalability of W-MSA for processing large-scale images.

### 3.2 Enhanced features representation block

A neural network block combining Depthwise and Pointwise Convolutions leverages Depthwise Convolutions (Guo et al., 2019) to capture spatial features independently across each channel, enhancing spatial granularity. The Pointwise Convolution (Hua et al., 2018) then integrates these spatially focused features, creating a rich, channel-combined representation that enhances the model’s ability to capture essential and discriminative features efficiently. Depthwise Convolution where each filter is applied to only one input channel. In contrast to standard convolutions, depthwise convolutions reduce computational complexity as given in Equation 7.


$$X_{v}(m,n)=\sum_{i,j}Y_{v}(m+i,n+j)\cdot W_{v}(i,j)$$ (7)

where $Y_{v}$ is the v^th^ channel of the input, $W_{v}$ is the depthwise filter for that channel, and $X_{v}$ is the corresponding output. This is a standard 2D convolution applied after the depthwise convolution. It combines the output from the depthwise convolution across channels as shown in Equation 8.


$$O(m,n)=\sum_{v}X_{v}(m,n)\cdot V_{v}$$ (8)

Where $V_{v}\;$ represents the filters in this conv2D layer applied across the depthwise operator. GELU is a smooth activation function, where the output is a stochastic binary decision with some non-linearity as shown in Equations 9, 10.


$$\text{Gelu}(y)=y\cdot P(Y\leq y)=y\cdot \frac{1}{2}(1+e(\frac{y}{\sqrt{2}}))$$ (9)

$$\hat{y}=\frac{y-\beta }{\gamma }$$ (10)

**Figure 3:**
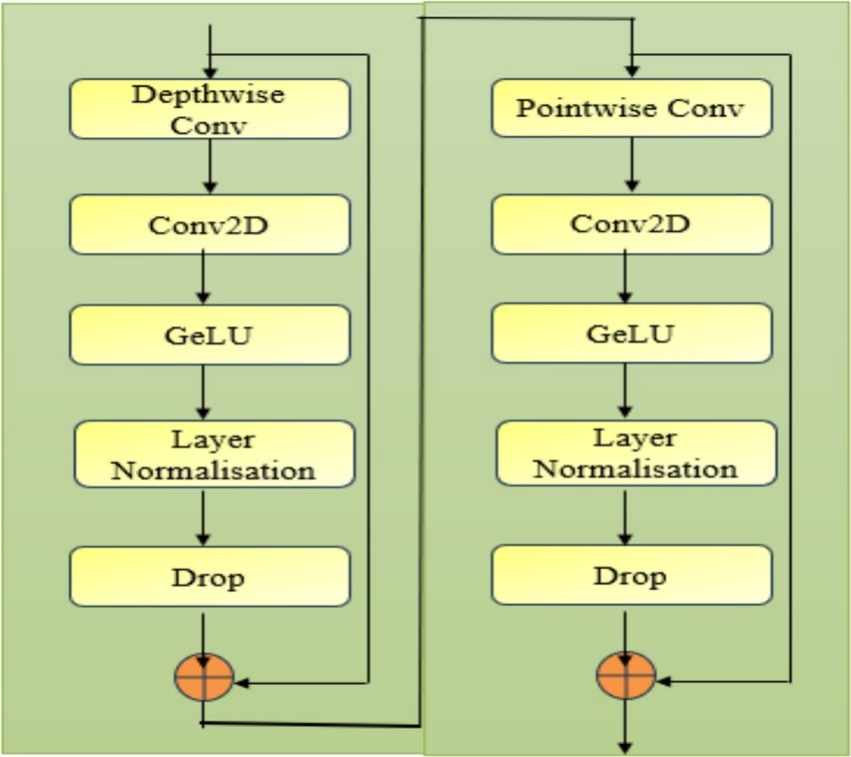
Overview of the enhanced feature representation model.

This process normalizes the activations across the features within a layer to improve stability and training efficiency. β refers mean and γ represents standard deviation within a layer. The output of the depthwise convolution branch is added back to the input via a residual connection, helping in training deeper networks by avoiding vanishing gradient issues. Pointwise Convolution is a 1×1 convolution applied across the channels, used to fuse information across channels without altering the spatial dimensions as given in Equation 11.


$$X_{p}(m,n)=\sum_{v}Y(m,n,v)\cdot W(1,1,v)$$ (11)

where v refers the channels in the input and the pointwise convolution across all channels. Conv2D, GELU, Layer Normalization, Dropout follow the same principles as the depthwise convolution branch. The Conv2D is used to mix information across channels after the pointwise convolution. GELU activation, Layer Normalization, and Dropout work identically in both branches to introduce non-linearity, normalize activations, and prevent overfitting, respectively. Similar to the depthwise branch, the output from the pointwise convolution branch is added back to the input. The depthwise convolution enahances the spatial features whereas Pointwise convolutions combine features across channels efficiently. The residual connections helped to overcome the vanishing gradients, and the normalization layers stabilized the learning process effectively. Layer Normalization and Dropout layers help improve the model’s generalization by stabilizing training and reducing overfitting, respectively. Figure 3 details the layers included in the enhanced feature representation model.

### 3.3 Optimised feature selection

Population-based optimization techniques use random searches to identify the optimal solutions. Also, an adaptive local search technique called Adaptive *β*-Hill Climbing (AβHC) is used to fine-tune the selected feature. This feature forms a mapping to the output classes, resulting in the Hierarchical Deep Learning Classifier (HDLC) to effectively distinguish between cracked and non-cracked surfaces based on the refined input features. The Sine-Cosine Algorithm (SCA) is a population-based metaheuristic used for feature selection and optimization (Mirjalili, 2016). It uses sine and cosine functions in an iterative process with two phases: exploration, which introduces diverse solutions to search broadly, and exploitation, which fine-tunes solutions by reducing randomness. Equation 12 defines how positions are updated using these functions.


$$C_{i,j}^{m+1}=\{\begin{array}{c} C_{i,j}^{m}+k_{1,j}^{m}\times \sin(k_{2,j}^{m})\times |k_{3,j}^{m}N_{j}^{m}-C_{i,j}^{m}|,k_{4,j}^{m}<0.5\\ C_{i,j}^{m}+k_{1,j}^{m}\times \cos(k_{2,j}^{m})\times |k_{3,j}^{m}N_{j}^{m}-C_{i,j}^{m}|,k_{4,j}^{m}\geq 0.5 \end{array}\}$$ (12)

Here, $C_{i,j}^{m}\;$ is the position in $j^{\mathrm{th}}$ dimension of $i^{\mathrm{th}}$ search element at $m^{\mathrm{th}}$ iteration. $k_{2,j}^{m},k_{3,j}^{m}\;\text{and}\;k_{4,j}^{m}\;\mathrm{are}\;$ random numbers, $N_{j}^{m}$ represents the position of $j^{\mathrm{th}}$ best solution at $m^{\mathrm{th}}$ iteration and || denotes the absolute value. A random value $k_{1,j}^{m}\;$ enables the transition from exploration to exploitation as shown in Equation 13.


$$k_{1,j}^{m}=\beta -m\frac{\beta }{M}$$ (13)

**Figure 4:**
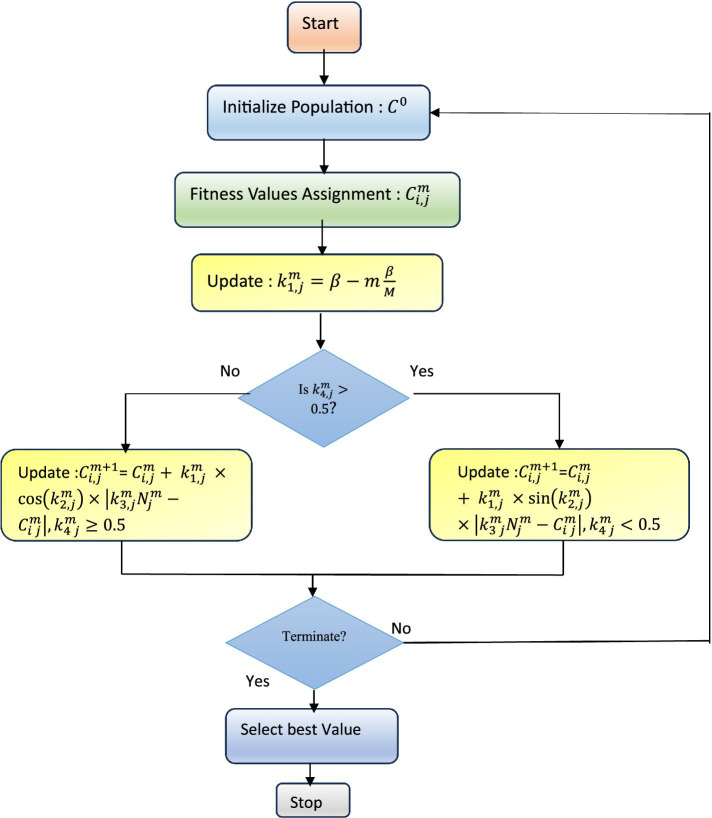
The flowchart for the population-based optimization algorithm.

Here, β and M characterize the constant value, and iterations, respectively. $\;k_{1,j}^{m}\;$ decides whether the search region is for $\left(k_{1,j}^{m}\in [-1,1]\right)$ or exploration $(k_{1,j}^{m}\in [-1,2])\;$ or $\left(k_{1,j}^{m}\in [1,2]\right)$ . The stochastic variable $k_{2,j}^{m}\;$ , ranging within [0,2π] , controls the search agent’s direction relative to the destination, aligning with the sine and cosine cycle. $k_{3,j}^{m}\;$ balances exploration and exploitation by assigning a random weight between 0 and 2, influencing step size—greater than 1 emphasizes, while less than 1 de-emphasizes the destination’s impact. $k_{4,j}^{m}$ manages the switch between sine and cosine functions, as outlined in Equation 14. The SCA feature optimization process is summarized in the flowchart shown in Figure 4. In order to enhance the exploitation ability Adaptive 𝛽-Hill Climbing (A𝛽HC) is integrated that utilizes local search-based techniques using two control parameters and $\;N_{\mathrm{hc}}$ and $B_{\mathrm{hc}}$ . The parameter $\;N_{\mathrm{hc}}$ is assigned close to 1 value which gradually decreases as the search process progresses. This permits the process to dynamically adjust $\;N_{\mathrm{hc}}$ to advance the search performance, as given in Equation 14.


$$N_{\mathrm{hc}}^{m}=1-\frac{m^{\frac{1}{p}}}{M_{\max}^{\frac{1}{p}}}$$ (14)

Here, $N_{\mathrm{hc}}^{m}$ denotes $\;N_{\mathrm{hc}}\;$ at time m, P linearly decrease the $\;N_{\mathrm{hc}}$ to a value close to 0 and $M_{\max}$ represents the upper limit B parameter adapts a range $\in B_{\mathrm{hc}}^{\min},B_{\mathrm{hc}}^{\max}\;$ , mathematically expressed in Equation 15.


$$B_{\mathrm{hc}}^{m}=B_{\mathrm{hc}}^{\min}+m\times \frac{B_{\mathrm{hc}}^{\max}-B_{\mathrm{hc}}^{\min}}{M_{\max}}$$ (15)

Here, $B_{\mathrm{hc}}^{m}$ represents the rate of $\;B_{\mathrm{hc}}$ at iteration m , with $B_{\mathrm{hc}}^{\min}\;\text{and}\;B_{\mathrm{hc}}^{\max}\;$ indicating the minimum and maximum value of $\;B_{\mathrm{hc}}\;$ respectively, $M_{\max}\;$ denotes the total number of iterations, and m refers to the current iteration. Figure 4 details the flowchart for the Population-based optimization algorithm (Raghaw et al., 2024) detailing the selection process of the optimized features.

## 4 Experimental results and discussion

Overview of the dataset, data augmentation methods, experimental setup, model training, and validation. It also details the performance metrics used to analyse the proposed model is detailed in this section.

### 4.1 Description of the dataset

**Figure 5:**
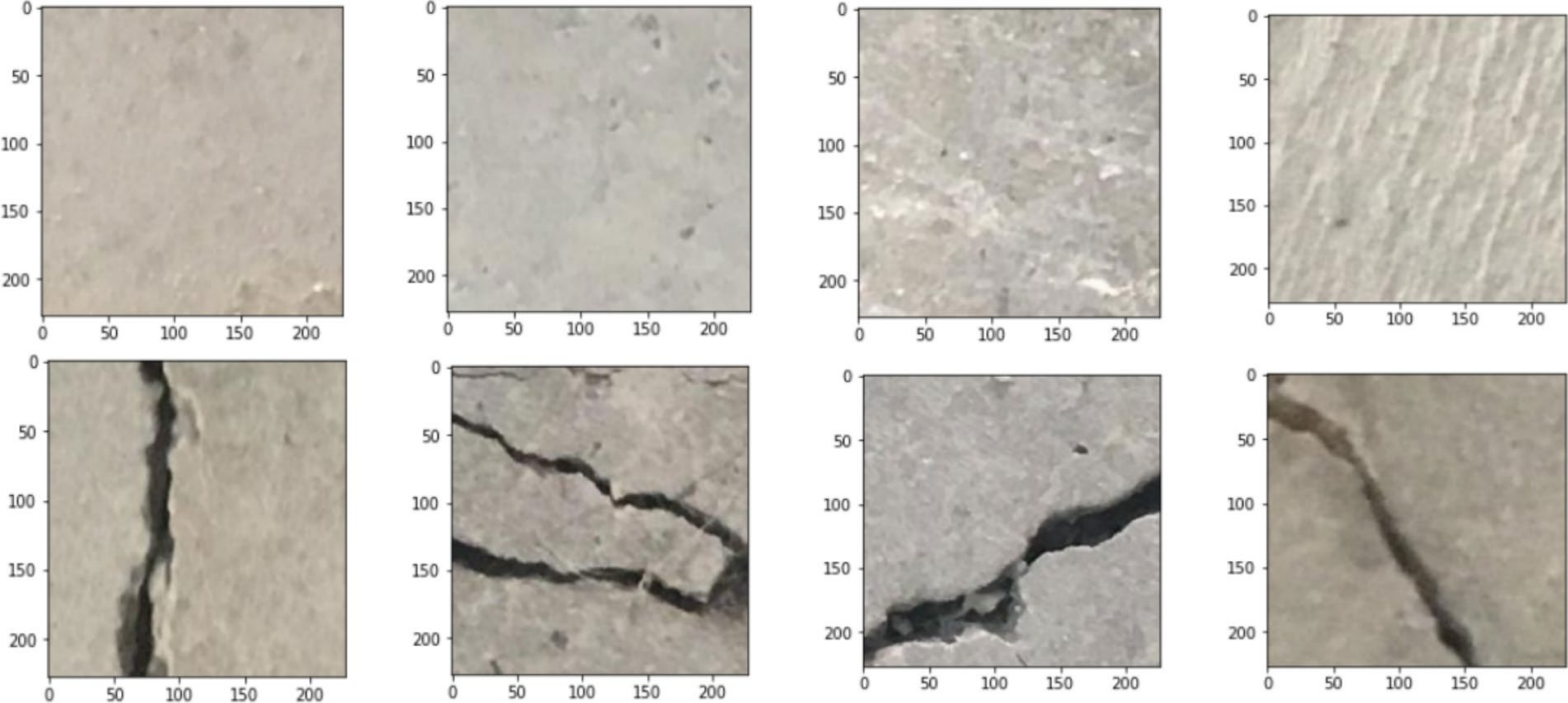
Sample images for both crack and non-crack images.

Concrete surface cracks is a defect commonly identified in civil infrastructure. Building inspection is crucial for assessing the structural integrity and tensile strength of these constructions. Crack detection (Özgenel, 2019; Özgenel and Gönenç Sorguç, 2018) plays a vital role in this process by identifying structural flaws and evaluating the overall condition of the building. These images are organized into two class: negative (no cracks) and positive (with cracks), suitable for image classification tasks. Each category includes 20,000 images, resulting in a total of 40,000 RGB images, each with a resolution of 227 × 227 pixels. The dataset was developed from 458 high-resolution images (4,032 × 3,024 pixels) following the method introduced by Zhang et al. (2016). These high-resolution images display considerable variation in surface texture and lighting conditions. Figure 5 shows few sample images for each class.

### 4.2 Environmental setup

The proposed model was implemented using PyTorch, an open-source deep learning framework. To optimize the model and minimize loss, the Adam optimizer was incorporated with a learning rate set to 0.0001. The training was performed on an Azure virtual machine powered by an NVIDIA Tesla P40 GPU.

### 4.3 Evaluation metrics

The metrics that are used to evaluate the model are as given in Equations 16–20. Accuracy measures how the predicted values are similar with the actual values. Precision identified true positive values. Specificity indicates the model’s ability to correctly identify true negatives, computed as the ratio of true negatives to the total number of negative cases. Recall represents the proportion of correctly predicted positive cases out of all actual positive data in the dataset. The F1 score, which is the harmonic mean of precision and recall, which reflects the model’s effectiveness in detecting positive samples. These evaluation metrics are calculated based on True Positives (TP), False Positives (FP), True Negatives (TN), and False Negatives (FN), as given in Equations 8–12.


$$\text{Accuracy}=\frac{\mathrm{TP}}{\mathrm{TP}+\mathrm{TN}+\mathrm{FP}+\mathrm{FN}}$$ (16)

$$\text{Precision}=\frac{\mathrm{TP}}{\mathrm{TP}+\mathrm{FP}}$$ (17)

$$\text{Recall}=\frac{\mathrm{TP}}{\mathrm{TP}+\mathrm{FN}}$$ (18)

$$\text{Specificity}=\frac{\mathrm{TN}}{\mathrm{TN}+\mathrm{FP}}$$ (19)

$$F1\;\text{Score}=\frac{2*\text{Precision}*\text{Recall}}{\text{Precision}+\text{Recall}}$$ (20)

### 4.4 Training details

The model included a 2 × 2 patch size for the initial image segmentation and processes these patches with 8 attention heads and a 64-dimensional embedding. The attention mechanism is performed within a 2 × 2 window which incorporates a shifted window of size 1. Dropout is applied with 0.03 to avoid overfitting. The training is carried out with a learning rate of 1e-3, batch size of 16, and 30 epochs, utilizing weight decay and label smoothing for improved generalization. The model used 80:20 ratio ensuring a suitable split for evaluating performance during the training process.

### 4.5 Ablation studies

This study evaluates the efficiency of major components in the proposed architectural framework that helps to optimize the performance. The efficiency of the following levels was evaluated: Swin Transformer, Enhanced Features Representation Block and Population based Optimisation for Feature Selection.

#### 4.5.1 Analysis of the Swin Transformer

**Figure 6:**
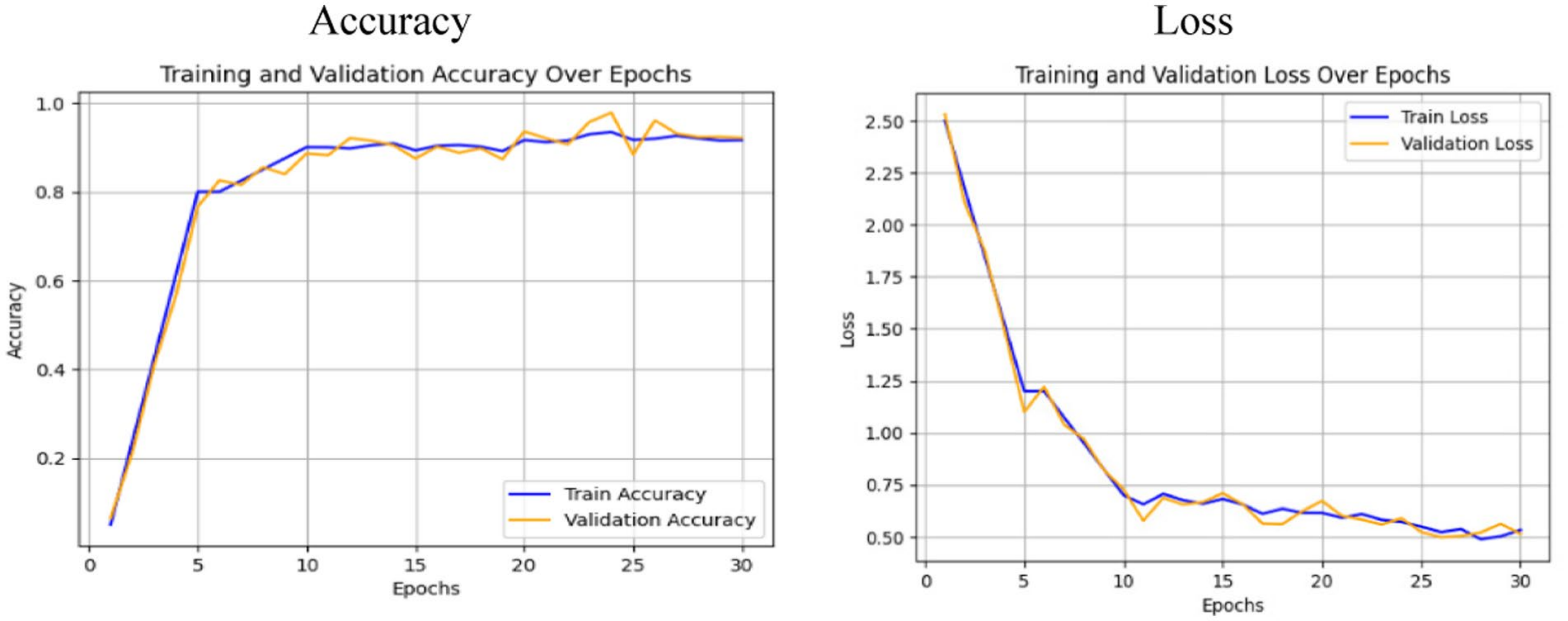
Accuracy and loss plot analysis obtained using Swin Transformer.

**Figure 7:**
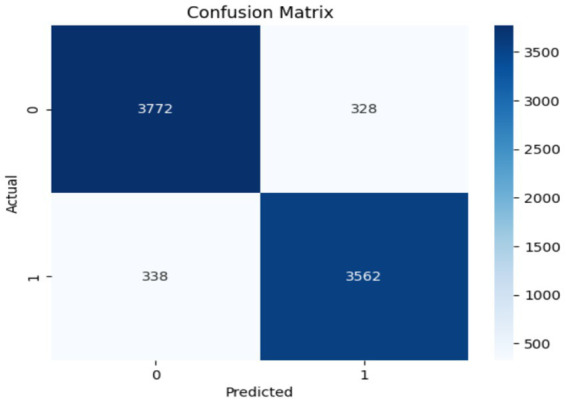
Confusion matrix obtained using Swin Transformer.

The performance of the Swin Transformer was analyzed as an independent module to evaluate its effectiveness in feature extraction for crack detection. This analysis shows the model’s ability to capture long-range dependencies, for identifying fine-grained crack patterns and irregularities in structural images. The Swin Transformer employed a hierarchical architecture with shifted windows, enabling efficient computation while preserving spatial granularity. The self-attention mechanism ensures robust modeling of both local and global contextual relationships, important for distinguishing cracks from background textures. Figures 6, 7 illustrate the training and performance metrics of the Swin Transformer block achieving a testing accuracy of 91.68%. The Swin Transformer’s performance as an independent module was further analyzed to assess its capability in crack detection. The training and validation curves showed rapid convergence within the first few epochs, achieving a stable testing accuracy of 91.68% with minimal overfitting. The confusion matrix indicated a strong balance between precision and recall for both crack and non-crack classes, demonstrating the model’s robustness in distinguishing fine-grained crack patterns from background noise. This performance highlights the Swin Transformer’s ability to effectively model both local and global contextual features through its hierarchical shifted window mechanism while maintaining computational efficiency, making it a reliable backbone for structural crack detection tasks.

#### 4.5.2 Analysis of the Swin Transformer with enhanced features representation block

**Figure 8:**
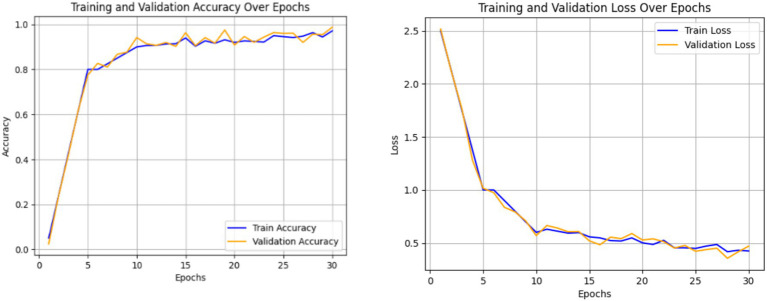
Accuracy and loss plot obtained using Swin Transformer and the EFRB.

**Figure 9:**
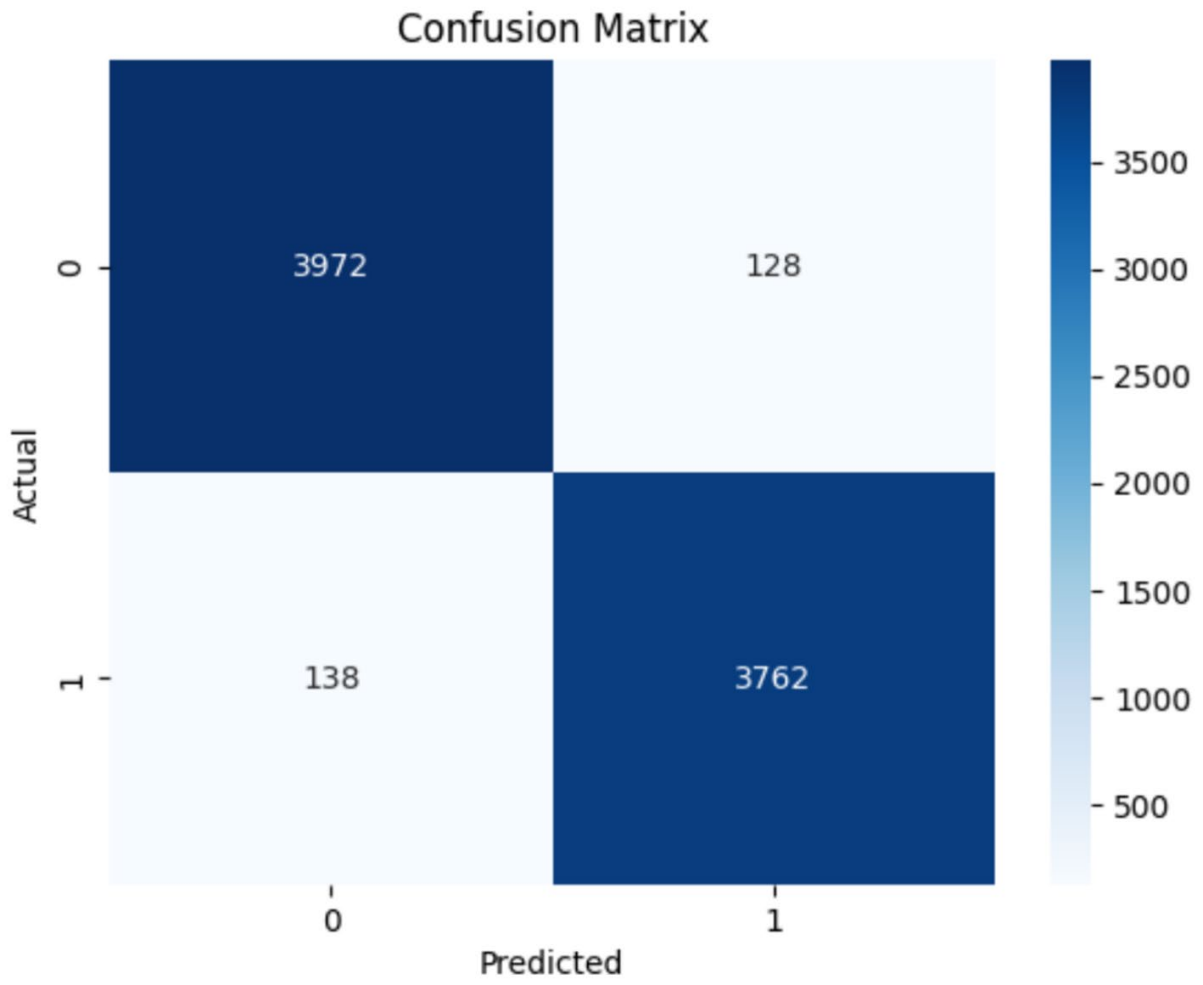
Confusion matrix obtained using Swin Transformer and the EFRB.

The combined performance of the Swin Transformer and the Enhanced Features Representation Block (EFRB) was analyzed to evaluate their collaboration in feature extraction for crack detection. The Swin Transformer is integrated with the EFRB’s ability to enhance spatial granularity and channel-wise representation, resulting in a feature extraction framework. The Swin Transformer acts as the initial stage, effectively processing complex input images with its hierarchical architecture and shifted window self-attention mechanism. This enables both global and local contextual relationships critical for detection of subtle and irregular crack patterns. The extracted features are then passed to the Enhanced Features Representation Block, which employs Depthwise Convolutions to independently refine spatial features across channels and Pointwise Convolutions to fuse these features into channel-combined representation. The EFRB’s residual connections and Layer Normalization stabilize training, while the GELU activation and Dropout layers prevent overfitting, ensuring robust learning. Figures 8, 9 illustrate the training process and performance evaluation of the Swin Transformer combined with the EFRB. The metrics demonstrate improved convergence rates, stability, and feature extraction efficiency compared to using the Swin Transformer as a standalone component attaining 95.43% as accuracy.

The integration of the Swin Transformer with the Enhanced Features Representation Block (EFRB) demonstrated improved feature extraction performance for crack detection. The Swin Transformer efficiently modeled both global and local dependencies through its hierarchical shifted window mechanism, while the EFRB enhanced spatial granularity and channel-wise representation using Depthwise and Pointwise Convolutions. Residual connections, Layer Normalization, and GELU activation stabilized training and reduced overfitting, ensuring robust learning. The training and validation curves showed faster convergence and higher stability compared to the Swin Transformer alone, while the confusion matrix confirmed significant improvement in classification accuracy, achieving 95.43%, highlighting the combined model’s effectiveness for precise crack detection.

#### 4.5.3 Performance analysis of the proposed model

The proposed model integrates the Swin Transformer, the Enhanced Features Representation Block (EFRB), and Population-based Optimization for Feature Selection, resulting an enhanced framework for crack detection. Each component contributes distinct strengths enhancing the network’s overall performance in extracting, refining, and selecting discriminative features. The Swin Transformer efficiently captures both global and local dependencies through its hierarchical architecture and shifted window mechanism. The Enhanced Features Representation Block (EFRB) refines and enhances the extracted features. The Depthwise Convolutions within the EFRB specialize in spatial feature extraction by independently processing each channel, while the Pointwise Convolutions integrate these spatially refined features across channels. Residual connections, along with GELU activation, Layer Normalization, and Dropout, ensure stable training, robust gradient flow, and generalization, resulting in a rich and discriminative representation tailored for crack detection. Population-based Optimization for Feature Selection ensures that the most relevant and informative features are prioritized while redundant or non-contributory features are minimized. By leveraging evolutionary algorithms, this optimization step enhances the model’s predictive accuracy while reducing computational overhead, making the network efficient and scalable. Table 2 shows the accuracy attained by the ablation studies of each component of the proposed work.

**Table 2:** Performance obtained during ablation studies.

Architecture	Accuracy
Swin Transformer	91.68%
Swin Transformer with enhanced features representation block	95.43%
Proposed work	98%

**Figure 10:**
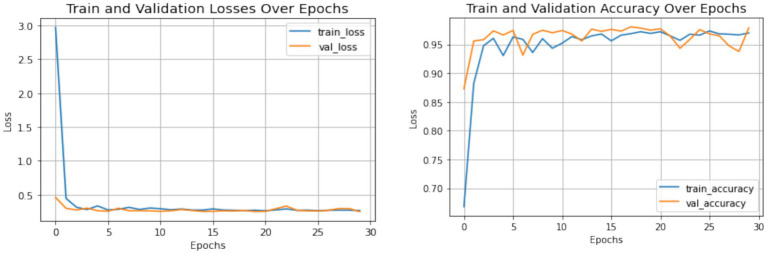
Accuracy and loss plot of the proposed model.

**Figure 11:**
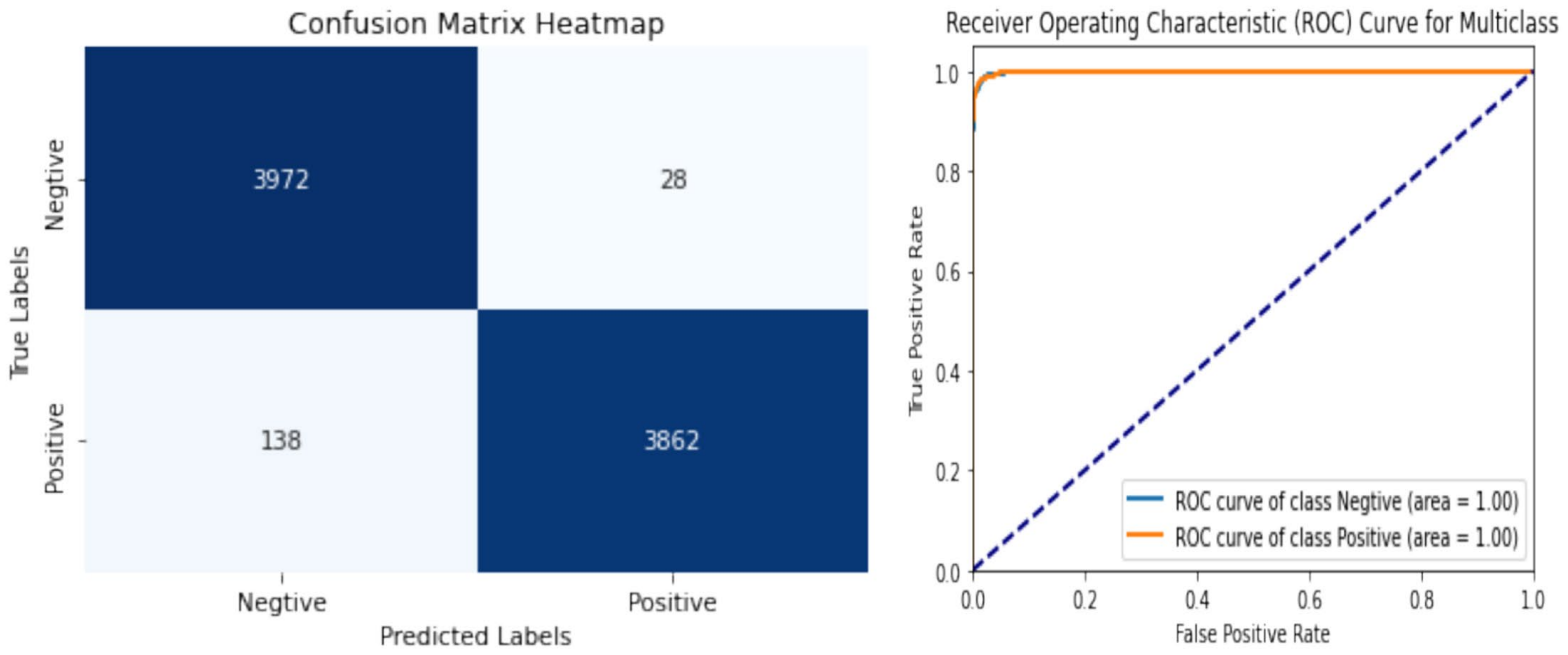
Performance metrics showing the confusion matrix along with the ROC plot.

Figure 10 illustrate the training process and performance of the proposed network. The results demonstrate a good improvement in accuracy, robustness, and convergence compared to individual components analyzed separately. Figure 11 shows the confusion matrix along with the ROC plot obtained for the proposed model. The integration of the Swin Transformer, EFRB, and Population-based Optimization ensures a powerful and balanced approach to crack detection, achieving high precision and generalization across diverse datasets.

The proposed network achieves enhanced performance with an overall accuracy of 98%, demonstrating precision, recall, and F1-scores of 0.97, 0.99, and 0.98 for crack detection, respectively, highlighting its robustness and effectiveness for real-world applications as shown in Table 3.

**Table 3:** Performance metrics of the proposed work.

Class	Precision	Recall	F1-Score	Sensitivity	Specificity
Crack	0.97	0.99	0.98	0.99	0.96
No Crack	0.99	0.97	0.98	0.96	0.99
Accuracy	98%				

The model, integrating the Swin Transformer, Enhanced Features Representation Block (EFRB), and Population-based Optimization, achieved a testing accuracy of 98%, significantly outperforming the Swin Transformer alone (91.68%) and its combination with EFRB (95.43%). The training and validation curves demonstrated rapid convergence and stable performance across epochs, while the confusion matrix confirmed high classification accuracy with minimal misclassification between crack and non-crack classes. Furthermore, the ROC curves for both positive and negative classes achieved an AUC of 1.0, indicating excellent discriminative capability.

### 4.6 Performance comparison with existing works

Elghaish et al. (2022) evaluated AlexNet, GoogleNet, and two others for highway crack identification and classification, and proposed a model optimized with diverse learning rates achieving 97.62% accuracy using a dataset of 4,663 crack images grouped into three categories, outperforming GoogleNet’s 89.08% and AlexNet’s 87.82%. Ahmadi et al. (2022) proposed a comprehensive approach combining image segmentation, noise reduction, heuristic-based feature extraction, and the Hough transform with crack classification using six classifiers. The hybrid model achieved the highest accuracy at 93.86%, surpassing individual classifiers. Liu et al. (2022b) employed infrared thermography and CNNs to classify asphalt pavement fatigue crack severity into four levels, using three image types. Thirteen CNN models, including EfficientNet-B4, were trained, with accuracy surpassing 0.95 across all image types, particularly on infrared images. Grad-CAM and Guided Grad-CAM analyses indicated fusion images are highly effective for reliable fatigue crack classification.

Liu et al. (2023) introduced a tunnel crack detection method using image processing with deep learning, comparing SVM and AlexNet-based models. AlexNet achieved 96.7% test accuracy, indicating deep CNN models’ superior performance for identifying structural flaws in subway tunnel. Chen et al. (2023) explored the role of deep learning, specifically transfer learning, in automating the detection of building facade cracks. Addressing the need for efficient large-scale inspections, transfer learning significantly improved CNN performance, increasing accuracy from 89% to 94%, demonstrating its efficacy in image classification with limited data, aligning with national Smart Nation goals for intelligent technology in construction.

Zhang et al. (2023a) presented a lightweight broad learning system for concrete crack detection, named MobileNetV3-BLS, which overcomes the challenges of complex architectures and high computational requirements. This method improved feature extraction by integrating MobileNetV3’s inverted residual structure as a convolutional module, employing random mapping and enhancement nodes to train the model. MobileNetV3-BLS exhibits enhanced accuracy and training speed, facilitating dynamic updates for incremental learning with new data and nodes. Table 4 provides a comparative analysis between the proposed model existing state-of-the-art architectures in crack detection.

**Table 4:** Comparison with state-of-the-art architectures.

Sl. No	Reference	Methodology	Accuracy in %
1	Elghaish et al. (2022)	Optimized CNN model with diverse learning rates vs. GoogleNet and AlexNet on 3-category crack dataset	97.62
2	Ahmadi et al. (2022)	Image segmentation + noise reduction + heuristic feature extraction + hybrid model (6 classifiers)	93.86
3	Liu et al. (2022b)	CNNs with infrared thermography and fused image types for fatigue crack severity classification	>95.00
4	Liu et al. (2023)	Image processing + deep learning (SVM vs. AlexNet) for subway tunnel crack detection	96.70
5	Chen et al. (2023)	Transfer learning for building façade crack detection	94.00
6	Zhang et al. (2023a)	MobileNetV3-BLS: lightweight broad learning with inverted residual structure and enhancement nodes	Not specified
8	Proposed	Swin+EFRP+Population based Optimization	98

## 5 Conclusion and future work

The proposed a crack detection framework integrating the Swin Transformer with an Enhanced Features Representation Block (EFRB) efficiently captured long-range dependencies and process complex images, while the EFRB improves spatial feature extraction and channel representation through depthwise and pointwise convolutions. Population-based feature selection optimizes the process, resulting an robust performance through effective exploration of the feature space. The proposed model achieved an accuracy of 98%, with precision, recall, and F1-scores of 0.97, 0.99, and 0.98, respectively, highlighting the model’s robustness in detecting cracks in real-world structural images. The results demonstrate the potential of combining advanced transformers with convolutional blocks for high-precision tasks in image analysis. The proposed framework can significantly enhance the accuracy and efficiency of crack detection systems, providing a valuable tool for structural monitoring and maintenance.

### 5.1 Future work

While the proposed model has shown strong performance, several avenues for future research can be explored. First, improving the model’s efficiency for real-time crack detection in large-scale datasets would be beneficial, potentially through model pruning, quantization, or more advanced techniques like knowledge distillation. Furthermore, exploring multi-modal crack detection by incorporating data from different sensors (e.g., thermal, acoustic) could improve the model’s robustness under diverse environmental conditions. Future work could also focus on extending the framework to 3D crack detection, enabling the model to handle complex, three-dimensional structural scans.

## Data Availability

The original contributions presented in the study are included in the article/supplementary material, further inquiries can be directed to the corresponding author. https://www.kaggle.com/datasets/arnavr10880/concrete-crack-images-for-classification.
